# Retraction: Occupational Stress and Insomnia Symptoms Among Nurses During the Outbreak of COVID-19 in China: The Chain Mediating Effect of Perceived Organizational Support and Psychological Capital

**DOI:** 10.3389/fpsyt.2024.1430791

**Published:** 2024-05-24

**Authors:** Frontiers Editorial Office

Following publication, concerns were raised regarding the ethical approval for this study. An investigation was conducted in accordance with Frontiers’ policies, and it was confirmed that the ethical approval was obtained after the study commenced. Lack of appropriate ethical approval is a breach of Frontiers’ guidelines and those of the Committee on Publication Ethics. As such, this article is retracted.

This retraction was approved by the Chief Editors of Psychiatry and the Chief Executive Editor of Frontiers. The authors agree to this retraction.

